# Victimization Perceived and Experienced by Teens in an Abusive Dating Relationship: The Need to Tear down Social Myths

**DOI:** 10.3390/healthcare11111639

**Published:** 2023-06-03

**Authors:** Isabel Cuadrado-Gordillo, Guadalupe Martín-Mora-Parra, Ismael Puig-Amores

**Affiliations:** Faculty of Education and Psychology, Department of Psychology and Anthropology, University of Extremadura, 06071 Badajoz, Spain; guadammp@gmail.com (G.M.-M.-P.); ipuigamores@unex.es (I.P.-A.)

**Keywords:** teen dating violence, male victimization, female victimization, moral disengagement, sexism

## Abstract

The phenomenon of adolescent dating violence is a social health problem that affects thousands of people in different contexts and parts of the world. To date, much of the work that has focused on analysing this phenomenon has tended to study it from the perspective of victimized adolescent girls, considering that gender violence predominates in violent pair relationships. Nonetheless, there is a growing body of evidence that the victimization of adolescent boys is a reality. Thus, mutual violence between boys and girls is increasingly prevalent. Given this context, the present study’s objective was to analyse and compare the victimization profile of a sample of female and male adolescents, taking into account the variables most commonly associated with victimization in these abusive relationships (perceived violence suffered, perceived severity, sexism, and moral disengagement). With this objective, different instruments were administered (CUVINO, Scale of Detection of Sexism Adolescents (DSA), and Mechanism of Moral Disengagement Scale (MMDS)). Data analysis based on the construction of a multiple linear regression model confirmed that the boys and girls in the sample revealed having suffered violence from their partners to a different degree. It is evident that the victimization profile of the two sexes is different. Thus, boys show less perception of severity, more sexism, and greater use of certain moral disengagement mechanisms than girls. These results reveal the need to tear down social myths and construct prevention programs that take into account different victimization profiles.

## 1. Introduction

Dating violence is one of the most common types of abuse among adolescents and young adults. These aggressions between dating couples begin at a very young age and can continue throughout life with different partners and relationships [1]. Thus, this phenomenon has been pointed to as one of the main factors associated with increased risks of abuse in adults [2].

The seriousness of the phenomenon has led to its global consideration as a social health problem of the first order and has encouraged the creation of various resources and programs for the care of victims, especially women. This fact highlights the general tendency to associate violence in pair relationships with the victimization of women. Gender violence based on control, abuse, and the development of asymmetric relationships is highly frequent throughout the world [3]. It has been pointed out that boys tend to show greater acceptance of violence within the couple [4], making it more likely that they become aggressors [5]. Nonetheless, neither the definition of adolescent dating violence nor that of violence in intimate relationships between adults is limited to gender violence.

The World Health Organization [6] and the Center for Disease Control and Prevention [7] establish violence in intimate relationships as being any conduct that causes the victim physical, psychological, or sexual harm, regardless of sex, gender, or the direction of aggressions [8]. With this, either member of the couple could in principle play the role of both victim and aggressor [9]. Evidence of male victimization in pair relationships has been found since the mid-1970s [10]. More recently, studies such as those of [11,12,13,14,15] have revealed victimization and bidirectional violence within the couple and a growing proportion of male victims [16], giving rise to different profiles of victimization and aggression in sentimental relationships.

There has been less research on male victimization than has been directed towards women. Some studies have pointed out, however, that men and women suffer similar attacks. For example, research conducted by the Spanish Government on violence in adolescent couples in 2015 revealed that both boys and girls tend to accept a certain degree of control by their partner [17], and this is also a common fact among adult couples [18,19]. Psychologists specialized in caring for adolescent girls victimized in a dating relationship point out that these young women often recognize that they perpetrate or have perpetrated aggression (especially control) towards their partners, largely copying the type of behaviour that boys display towards them [20].

However, control is not the only violent behaviour committed by female aggressors. It has been noted that girls’ acceptance of violence is related to their own physical aggression towards their partners [21]. In a study carried out by Hines and Douglas [19], 77.5% of the sample of men reported having suffered minor physical attacks by their partner in the previous year, while 35.1% claimed to have suffered serious injuries. Recent studies have confirmed the presence of male victims of physical violence, but in different proportions [22,23]. Likewise, various works have pointed not only to the high prevalence of psychological violence in abusive relationships but also to the reciprocity and symmetry in the aggressions committed between men and women [18,24,25,26,27]. Similar conclusions were revealed by other studies, such as those carried out by Sears, Byers, and Price [28], who indicated that 35% of men and 47% of women confessed to having perpetrated psychological aggression towards their partners. With respect to addressing sexual violence, some studies have also pointed out that male victimization is a result of non-consensual sexual assaults by their partners [19,29]. In young adult and adolescent couples, research on male victimization and mutual aggression has been even less extensive, although various authors have revealed its existence in different contexts and countries [30,31,32,33]. Recently, some authors have reported similar results to those found among adult couples. Those workers indicate the existence of up to four different profiles of victimization and aggression, as well as the presence of reciprocal violence in young couples [34]. The first of these profiles involves low-intensity aggression towards an adolescent partner, directed at both boys (54%) and girls (40%). This study revealed the existence of mutual aggressions of psychological (34% girls and 33% boys) and physical (14% girls and 5% boys) types. The last profile found comprised multiple victimizations involving various types of violence in 8% of the cases of boys, with mutual psychological violence and sexual victimization being more prevalent among girls (12%).

Despite the evidence that has been found, there have been few studies that have analysed the specific profile of male victimization beyond merely pointing out its existence and the type of violence exerted or suffered. This fact further complicates the characterization and localization of factors that explain the initiation and maintenance of abuse in the case of boys. In contrast, the victimization of adolescent girls in this phenomenon has been widely studied, and they have been linked to such factors as the idealized vision of love [35] partly as a consequence of the great diffusion and consumption of audiovisual products of popular culture (film, television, and music) from early childhood onwards. The said ideal vision gives rise to the normalization, justification, and tolerance of aggression when it is committed by the partner [36] such that they understand violence as a normal way of interacting between couples, resulting in important consequences. This makes it hard for the victim to identify the victimization itself and ask for help. At the same time, other relevant factors linked to violence in adolescent couples have been noted. Examples are gender stereotypes and ambivalent sexism [37], the use of moral disengagement mechanisms as a means to justify the aggressor’s conduct [38,39,40], the victim’s inactivity to seek help [41], a history of domestic violence [4,42], and peer approval or personal factors such as negative emotionality [43].

Knowledge of the victimization profile of male adolescents is essential not only for reducing the taboo existing around the recognition and visibility of male victimization in dating relationships [22,44,45] but also for designing adequate prevention and care measures for not only women but also for men, resources that have up until now been neglected [46]. In this context, the principal objective of this study was (i) to investigate the victimization profile of adolescent boys in violent dating relationships, taking into account some of the factors traditionally linked to victimization (perception of severity in different types of violence, sexism, and moral disengagement); (ii) locate possible risk factors; and (iii) compare the profile found in victimized boys with that of victimized girls of the same age. This comparison is carried out to shed light on and help characterize male victimization and also to improve understanding of the phenomenon of dating violence and mutual aggression between men and women within the context of early intimate relationships.

## 2. Materials and Methods

### 2.1. Sample

The sample of this cross-sectional study comprised a total of 2577 adolescent students between the ages of 14 and 19 (44.8% boys). Participants were selected randomly and proportionally by a stratified sampling process in different stages. The process focused on randomly selecting a group of students that included different educational levels. Thus, the participants are students of lower secondary (ESO), upper secondary (Baccalaureate), and higher education (university and professional training). These students are from different areas of the Extremadura Region, including its two provinces (Cáceres and Badajoz), in the southwest of Spain. The selected areas cover both urban and rural zones of the region, with different socioeconomic characteristics and levels. Thus, approximately half of the sample belong to families exhibiting a medium–high level of purchasing power, and the other half exhibit a medium–low level of purchasing power. Similarly, the families had different academic levels, with half of the parents having completed higher education (Higher Level Education Cycles or University Studies) and the other half having completed secondary studies (Intermediate Training Cycles or Baccalaureate Certificate) or basic education.

In each of the schools selected, the questionnaires were administered collectively from the 3rd year of ESO to the 2nd year of Baccalaureate. The participants studying higher education were in their first year of university or the first year of their professional training. Their ages depended on their academic level (3rd ESO: 14–15 years; 4th ESO: 15–16 years; 1st Baccalaureate: 16–17 years; 2nd Baccalaureate: 17–18 years; university and 1st year of professional training: 18–19 years).

### 2.2. Instruments

Dating Violence Questionnaire, CUVINO [47]: This questionnaire has a total of 61 items, and they are grouped into two different blocks. The first addresses the types and incidences of violence that adolescents have received from their partners as well as the perception of severity they have regarding abusive behaviour. There are eight modalities of aggression considered for both frequency and perception of severity: detachment (“Is a good student, but is always late at meetings, does not fulfill his/her promises, and is irresponsible”), humiliation (“Ridicules your way of expressing yourself”), sexual (“You feel forced to perform certain sexual acts”), coercion (Threatens to commit suicide or hurt himself/herself if you leave him/her”), physical (“Has thrown blunt instruments at you”), gender (“Has ridiculed or insulted women or men as a group”), emotional punishment (“Refuses to give you support or affection as punishment”), and instrumental punishment (“Has stolen from you”).

The first block uses a five-point anchor Likert-type scale with five anchor points ranging from “never” to “almost always”. The reliability analysis (Cronbach’s alpha) obtained from the sample of this study produced values ranging from 0.66 to 0.83 for the envisaged types. On the other hand, the perception of severity uses a five-point Likert-type scale with 1 being “a lot” and 5 being “not at all”. The reliability for these modalities ranged from 0.71 to 0.84.

The second block focuses on victimization and the perception teenagers have of their characteristics as victims (duration of the relationship, attempts to break up, actual relationship with the aggressor, etc.).

Scale of Detection of Sexism in Adolescents (Escala de Detección de Sexismo en Adolescentes, DSA): This scale assesses the sexist attitudes that adolescents have towards traditional gender traits and roles, including two scales—hostile sexism and benevolent sexism [48]. Additionally, it has two secondary scales—sexist traits associated with F/M and the ability of each sex to perform roles and functions. The scale features six Likert-type points ranging from “totally disagree” to “totally agree”. The reliability analysis (Cronbach’s alpha) produced a value of 0.89 for the instrument overall, 0.91 for the hostile dimension, and 0.85 for the benevolent dimension.

Mechanisms of Moral Disengagement Scale (MMDS) [49]: This last questionnaire has a total of 32 items. These items allow obtaining 8 partial scores: moral justification, euphemistic language, advantageous comparison, displacement of responsibility, distortion of responsibility, distortion of consequences, attribution of blame, and dehumanization. The scale is a 5-anchor Likert-type scale ranging from “Strongly disagree” to “Strongly agree”. The internal consistency of the test (Cronbach’s alpha) is 0.74, and the reliability of the 8 mechanisms ranges between 0.72 and 0.81.

### 2.3. Procedure

Prior to the distribution of the questionnaires to the adolescents, both the research objectives and the procedure, instruments, and techniques used were checked and approved by the Bioethics and Biosafety Committee of the University of Extremadura (Spain) (Ref. 18/2017). The second step of the research study consisted in obtaining authorization from the Regional Educational Administration, and the project and its objectives were presented. Later, the management teams of lower and upper secondary schools were approached to invite them to participate in the study, describing the objectives and purpose of the study and the use of the data and ensuring the privacy and anonymity of adolescents. The written invitation was followed by personal telephone calls to the head teacher of each school to coordinate the collection of data covering the different levels (day and time of data collection).

Once authorizations to enter schools had been obtained, parental approval was also requested, considering that most participants were minors, by means of a document describing the nature of the study, the objectives, and the mechanisms used to guarantee the anonymity and confidentiality of the collected data. The letter was accompanied by an authorization form that parents had to sign and send back to the school in order to authorize the participation of their children in the study. In the case of participants of legal age (university students or professional training), the purpose of the study and its objectives were explained directly to them, ensuring the privacy and anonymity of responses. Additionally, they were asked to sign an informed consent document.

After obtaining authorization, the questionnaires were distributed in hard copies and completed by the participants. All questionnaires were completed collectively in each class and educational level. The instructions given by the researchers were the same in every class (anonymity was ensured, the Likert-scale was explained, the minimum time for a relationship (about a month), etc.). Additionally, we stayed in the class while the students filled out different questionnaires in order to clarify any possible doubts they could have.

### 2.4. Data Analysis

The data were analysed using statistical software package SPSS vn 24. Preliminary analyses began with the identification of victimized adolescents. Subjects who scored 0 on the CUVINO questionnaire were considered non-victims of adolescent dating violence. Those who scored higher than 0 were considered victims and assigned to two different victimization groups based on the frequency with which they had undergone the attacks: those who scored less than 3 were assigned to the “Sometimes” victim of violence group, and those with scores greater than 3 were classified as “Frequent” victims.

Secondly, the incidence of perceived violence suffered, the perception of the severity of the violent behaviours, the degree of sexism (hostile sexism, benevolent sexism, sexist trait, and sexist attitude), and the use of moral disengagement mechanisms (moral justification, euphemistic language, advantageous comparison, displacement of responsibility, distortion of consequences, attribution of blame, and dehumanization) were determined. The descriptive statistics of the study variables were computed, and Student’s *t*-test was used to compare each dimension by sex. The strength of the associations was measured using Cramér’s V and the chi-squared test.

Finally, the variables associated with the risk of violence were analysed by means of stepwise multiple linear regression, and these included the total score obtained with respect to violence as the dependent variable; the sex of the victims and different dimensions of the scales used during data acquisition (perceived severity of violent behaviour, sexism, and moral disengagement mechanisms) were used as predictor variables.

## 3. Results

The preliminary analyses revealed that, of the total sample of 2577 adolescents, 1232 had suffered violence on some occasions. Of these, 1190 adolescents are classified as victims who have suffered dating violence “sometimes” (sometimes frequently in CUVINO), and 42 perceive themselves as “frequent” victims (usually almost always in CUVINO); thus, the number of victims decreases as the frequency of violence increases. In both groups, the number of female adolescent victims is greater (Table 1).

The analysis of the CUVINO questionnaire, which directly asks the participants if they have ever felt mistreated (item 45), shows that, despite the fact that boys and girls indicate having suffered violence to a different extent, only 22 boys identify themselves as victims of abuse, while 69 girls report having been mistreated in a dating relationship.

With respect to the types of violence suffered, male and female victims coincide in that the most prevalent types are detachment, coercion, and emotional punishment. The analysis of associations between the variables, as measured using the chi-squared and Cramér’s V statistics, reveals that there is a statistically significant relationship between the sex of the victims and coercion incidence (*χ*^2^ = 39.91; *V* = 0.180; *p* < 0.01), emotional punishment incidence (*χ*^2^ = 38.242; *V* = 0.176; *p* < 0.001), and instrumental punishment incidence (*χ*^2^ = 21.49; *V* = 0.132; *p* < 0.01) (Table 2). Thus, although the three most frequent types of violence are identical in male and female victims, boys perceive a greater degree of violence suffered in these three types of abuse.

With regard to the victims’ perception of the severity of different types of violence, there are differences in the aggression that boys and girls consider as most serious. Specifically, boys point to physical violence severity (X = 4.10; SD = 1.19), humiliation severity (X = 4.06; SD = 1.19), and instrumental punishment severity (X = 4.04; SD = 1.16), and girls point to physical violence severity (X = 4.62; SD = 0.89), humiliation severity (X = 4.50; SD = 0.79), and sexual violence severity (X = 4.49; SD = 0.84). Likewise, the analysis of associations between the variables using the chi-squared statistic shows a statistically significant association between the perception of severity with respect to all types of violence and the victim’s sex. Being male or female is linked to the importance given to different types of violence, with the latter perceiving greater severity in all types of analysed victimization.

The results of the sexism questionnaire show differences between the degree of sexism of male victims and female victims in the four analysed variables. Adolescent girls score lower than boys on the four subscales, thus showing a lesser degree of sexism than boys. This fact is especially significant in the case of hostile sexism and the attitude that the participants show towards the capacity of men and women to carry out different roles and social functions. In this sense, an association is found between the sex of adolescent victims and the dimensions “hostile sexism” (*χ*^2^ = 0.37. *p* < 0.001) and “attitude towards performance of roles and functions” (*χ*^2^ = 0.36; *p* < 0.001) (Table 3).

The analysis of the moral disengagement scale once again points to differences in the use that boys and girls make of these mechanisms. Thus, descriptive statistics reveal that the resources most used by boys are “moral justification” (X = 2.01; SD = 0.55), “advantageous comparison” (X = 1.63; SD = 0.49), and “displacement of responsibility” (X = 1.59; SD = 0.46). The victimized adolescent girls, to a greater extent, used “moral justification” (X = 1.68; SD = 0.45), “displacement of responsibility” (X = 1.55; SD = 0.43), and “advantageous comparison” (X = 1.44; SD = 0.38). Hence, while they use the same mechanisms, their relative importance is different, with victimized boys also indicating a higher frequency of use. In sum, there is a statistically significant association between the sex of the victims and the use of the seven moral disengagement mechanisms (Table 4).

To determine whether there are differences by sex in the study’s variables, an analysis of differences was performed using Student’s *t*-test (Table 5). There were statistically significant differences in the factors “perception of severity” (*t*_1015.358_ = −13.31; *p* < 0.001), “hostile sexism” (*t*_1072.025_ = 6.39; *p* < 0.001), “attitude of each sex to performance of roles” (*t*_1040.804_ = 6.42; *p* < 0.001), “moral justification” (*t*_1032.755_ = 12.01; *p* < 0.001), “euphemistic language” (*t*_894.995_ = 10.44; *p* < 0.001), “advantageous comparison” (*t*_1031.426_ = 6.95; *p* < 0.001), “ diffusion of responsibility” (*t*_1045.414_ = 2.22; *p* < 0.05), “distortion of consequences” (*t*_900.854_ = 6.49; *p* < 0.001), “victim blaming” (*t*_966.027_ = 7.22; *p* < 0.001), and “dehumanization” (*t*_962.802_ = 7.77; *p* < 0.001).

For the factor “negative perception of severity”, the girls’ mean scores are higher than the boys’, but for all moral disengagement mechanisms, the contrary is the case. Nonetheless, the calculated effect sizes for most constructs are very small (<0.20). The exceptions are “perception of severity”, “hostile sexism”, “fitness of each sex to perform roles and functions”, “moral justification”, “euphemistic language”, and “dehumanization” (moderate effect sizes between 0.20 and 0.70). It could be considered that, although significant, the contribution is not relevant in the rest of the variables.

Finally, stepwise multiple linear regression was used to analyse the variables that were associated with the perceived violence suffered. The dependent variable was the perceived violence suffered. The predictor variables were the dimensions of the administered scales (perception of severity with respect to the different types of violence, hostile sexism, benevolent sexism, sexist attitude, sexist trait, and moral disengagement mechanisms), adding the sex of the participants and the age as control variables in order to eliminate its effect from the model. The resultant models had no problems with respect to multicollinearity (VIF < 5), and the percentages of variance explained ranged from 10% to 11%.

Table 6 lists the results of the two models. In Model 1, the male victims’ perceived violence that they have suffered is predicted, to the greatest extent, by annoyance with the “humiliation” type of violence. In Model 2, the female victims’ perceived violence that they have suffered is predicted to the greatest extent by annoyance with the “instrumental punishment” type of violence (Table 6).

## 4. Discussion

The results found in this study reveal the high presence of victimization in adolescent dating relationships, with both boys and girls being subjected to victimization. Thus, it was observed that the violence suffered in the first dating relationship, far from being confined to women, equally affects young men. Adolescent boys suffer abuse in their romantic relationships both sporadically and frequently, similarly to what was found in relation to female victimization. This fact seems to point to the high prevalence of mutual violence in abusive relationships, following the same pattern as found in recent research with adults [50]. With this, victimization in adolescent dating would not only be one of the main factors that predict violence between adult couples but would also be one of the main predictors of reciprocal violence in such relationships.

In contrast, the results reveal that boys show a lower degree of perception of severity than girls in all the types of analysed violence. While the girls perceive violence as serious, the boys show less rejection of abuse, downplaying violent behaviour to a certain extent. In relation to this, various studies [51,52] have shown that men who tend to accept violence are those who also tend to commit more acts of abuse towards their partner, thus adopting the role of the aggressor more easily. Nonetheless, this acceptance of dating violence could also cause boys, even when they are the ones who suffer from this type of violent behaviour, to view it as less important.

Based on the above, there is a fundamental difference between adolescent boys and girls victimized in a relationship. While girls normalize the violence perpetrated by their partners as being a result of factors such as the idealization of love [53] or the presence of latent benevolent sexism [37], they continue to be aware of the seriousness of the abuses even when they have normalized them [54]. With this, it is possible that prevention campaigns and public health interventions that are focused on the empowerment of women and on the modification of the structural environment of women [55], by highlighting the undesirability of violence, have had a positive effect on pair relationships. They seem to have failed, however, to get young women to identify their own victimization, ultimately prolonging the maintenance of violence in dating [56].

Boys may also accept violence, but for different reasons. In this sense, various authors have pointed to the taboo existing in society regarding the consideration of men as victims [22,44,45]. This fact may encourage mistreated adolescent boys to avoid interpreting the violence they receive as abuse, thus refusing to see themselves as victims. This may be of great importance to them with respect to protecting their self-esteem, but at the same time, it would prolong their victimization and their maintenance of the violent dating relationship. Additionally, it is possible that, from a masculine perspective, the abuses are classified as normal forms of relating to the partner, and the importance of the different types of abuse and aggressive behaviours is interpreted as being less serious. In this regard, it has been found that violence is better tolerated by men when the aggressors are women [57]. Likewise, some studies have found that supporting a patriarchal ideology, with the assumption of male and female stereotypes and sexual roles, encourages the growth of violence in pair relationships [58] and resistance to an awareness of victimization behaviours. These findings point in the same direction as the results of this present study: i.e., that adolescent boys present a greater degree of hostile sexism and sexist attitudes than girls. Both types of sexism seem to support the acceptance of violence within couples, especially for young men and women who tend to adopt the stereotype associated with their gender role.

While, according to the analysis of results, hostile sexism and sexist attitudes are associated with victimized boys, benevolent sexism appears in all victims regardless of their sex. These findings reinforce the presence of a high degree of normalization of certain types of violence in the sense that girls believe that they must be protected by men [59] while simultaneously romanticizing and tolerating this type of behaviour with the belief that it is carried with the aim of caring for them. The boys, for their part, would adopt this role of protector, thinking that they exhibit behaviours that are not classified as abusive because they fit the stereotype of the man as a caregiver [60].

The belief that women need affection and the assumption that, to be happy, every man needs a woman could create a form of sexism directed towards men and women that, although seemingly contradictory, would actually be interdependent and complementary [61]. Then, without being aware of it, the victimized adolescent boys might also be taking on the hostile sexist stereotypes of women as being weak and inferior [62] to justify girls’ violence, additionally believing that women do not have the capacity or ability to cause real harm with their violent behaviours.

Another relevant finding of the study is that victimized boys use moral disengagement mechanisms to a greater extent than girls. Other work too has suggested the existence of a consistent relationship between aggression and moral disengagement from the committed abuses [63]. Nonetheless, it seems that the use of these mechanisms is also important from the victim’s perspective. In the case of boys, this finding of a greater frequency of use may be linked to a twofold objective. One is that possible aggressions committed in the context of a dating relationship are justified as a form of achieving personal objectives [64] within that relationship. The other is that these mechanisms would also fulfil the function of justifying both the individual’s own position as a victim as well as the abuses perpetrated by their aggressors. The process followed would thus be similar to the justification that victims of bullying in school make with respect to the violent behaviour of their aggressors [65].

For their part, victimized girls might not need these moral disengagement tools to the same extent since they are already normalizing the violence; they normalize these situations as benevolent sexism and normalize violence via the use of the factors mentioned above. In this context, moral disengagement would be one more instrument combined with the rest of the factors that sustain victimization in the long term. The aggressions that girls commit towards their partners would not have such a marked need for moral justification since girls might see them as a right that they have in a social context of false empowerment [65,66], which places women in the same position as men. In this way, adolescent girls seem to tend to repeat the same type of maladjusted behaviour that they see in their aggressive partners, and this is a factor that fosters an increase in mutual aggression in adolescent dating relationships [20]. Similar results have been suggested in the contexts of bullying and cyberbullying in which victims of school violence tend to become aggressors in cyberspace, understanding these behaviours as a fully justified form of revenge [67,68,69] that provides some degree of compensation [70].

Differences that are found between the predictive models of the frequency of violence in boys and girls stand out. Firstly, the sex of the victim is not an important predictor variable. This reinforces the aforementioned idea of the great presence of mutual violence in adolescent dating relationships [30,31,32,33,34]. Then, there is the fact that although the boys share some of the same predictor variables with victimized girls, the model resulting for them includes a greater number of variables. The prediction of violence in the case of victimized boys includes variables such as hostile sexism and the perception of the severity of two specific and interrelated types of violence—humiliation and coercion. This fact is important, especially given that humiliation has been pointed out as a particularly serious type of abuse for men [71] due to its relationship with the social idea of masculinity [72]. Thus, authors such as those who published [73] have noted that men who feel they have been humiliated tend to respond violently to restore balance and preserve their reputation [74,75]. In recent studies, authors such as [71] have suggested the importance of deconstructing social myths around masculinity in order to prevent violent behaviour from being used as a means to demonstrate manhood. Thus, the assaults and abuse related to this specific type of violence might not only increase the victimization of boys in dating relationships but also foster a proportional increase in mutual aggression when the two partners try to restore balance, thus creating a downward spiral of abuse that teenage boys and girls find hard to escape.

On the contrary, in the case of girls, the regression model shows how the most important factor in predicting victimization is annoyance with the “instrumental punishment” type of violence. This finding could be related to the ideal vision of love, which increases the normalization, justification, and tolerance of aggression when it is committed by a partner [36]. This fact seems also to be linked with the use of moral justification as a tool to ignore abusive behavior [38,39,40].

## 5. Limitations

The study has some limitations that must be considered. First, it is cross-sectional. A longitudinal study could provide data on adolescents’ evolution and the possible changes that might arise in aggressive dating relationships. Such data would help delve deeper into the evolution and development of victimized adolescents and make it possible to identify additional variables to take into account that would undoubtedly be of interest in the design of short-, medium-, and long-term prevention programs. Likewise, a possible future line of research would be to incorporate the virtual context into studies of the phenomenon of violence in adolescent dating. As digital natives, a large proportion of the abusive control behaviours that boys and girls exhibit are carried out using instant messaging applications and social networks. An in-depth analysis of these behaviours would help contextualize the phenomenon within today’s information and communication society.

## 6. Conclusions

Teen dating violence has been studied extensively, but the field of adolescent male victimization has been less explored. The main contribution of the present study is its focus on male victims in an attempt to gain a broad and full understanding of the phenomenon. One of the main findings is that although boys perceive suffering different types of violence in their dating relationships, their perception of the severity of all types of analysed abuse is significantly lower than that indicated by female victims. In this way, the results show that boys give less importance to abuse than girls, even when they themselves are the victims. Nonetheless, neither boys nor girls seem to recognize their own victimization, leading to normalization and tolerance of violence that are motivated by different factors.

Other relevant findings indicate that boys present certain types of sexism more and exhibit a greater use of moral disengagement mechanisms than girls. The study thus reveals the importance of considering the sexism of victimized adolescents as an interdependent factor; in this case, the stereotypes that boys accept as their own encourage the stereotypes and roles that girls accept. Likewise, the use of moral disengagement mechanisms seems to have become another tool at the service of normalizing, justifying, and tolerating mutual violence in abusive relationships during adolescence. These discoveries have important practical applications. There is a need to put forward multidisciplinary strategies of prevention and intervention that are adapted to the characteristics of victims, and they should include boys in these interventions not only as possible aggressors but also as victims while considering the influence of cultural and social factors as well. Reciprocal violence needs to be recognized as a common problem in the phenomenon of adolescent dating violence.

## Figures and Tables

**Table 1:** Victimized adolescents.

	Sometimes	Frequently	Total
Male	500	18	518
Female	690	24	714
Total	1190	42	1232

**Table 2:** Statistical description of the CUVINO questionnaire.

	Male Victims		Female Victims			
	$\overset{-}{x}$	SD	$\overset{-}{x}$	SD	*χ^2^*	*V*
Detachment incidence	1.52	0.54	1.53	0.55	28.54	0.152
Humiliation incidence	1.25	0.38	1.23	0.38	10.73	0.093
Sexual incidence	1.23	0.44	1.19	0.40	18.79	0.124
Coercion incidence	1.43	0.46	1.39	0.53	39.91 **	0.180 **
Physical incidence	1.13	0.35	1.1	0.27	23.55	0.138
Gender incidence	1.22	0.35	1.25	0.50	16.98	0.117
Emotional Punishment incidence	1.41	0.55	1.31	0.51	38.242 ***	0.176 ***
Instrumental Punishment incidence	1.11	0.37	1.05	0.20	21.49 **	0.132 **
Detachment severity	3.61	0.97	4.35	0.71	199.00 ***	0.406 ***
Humiliation severity	4.06	1.19	4.50	0.79	223.86 ***	0.432 ***
Sexual severity	3.62	1.18	4.49	0.84	474.75 ***	0.630 ***
Coercion severity	3.62	0.97	4.25	0.81	219.14 ***	0.425 ***
Physical severity	4.10	1.19	4.62	0.89	198.33 ***	0.407 ***
Gender severity	3.62	1.18	4.41	0.84	272.73 ***	0.477 ***
Emotional Punishment severity	3.47	1.07	4.13	0.95	149.78 ***	0.353 ***
Instrumental Punishment severity	4.04	1.16	4.43	0.95	97.60 ***	0.286 ***

** *p* < 0.01; *** *p* < 0.001.

**Table 3:** Descriptive analysis sexism questionnaire.

	Male Victims		Female Victims			
	$\overset{-}{x}$	SD	$\overset{-}{x}$	SD	*χ^2^*	*V*
Hostile sexism	1.61	0.72	1.35	0.64	167.463 ***	0.37 ***
Benevolent sexism	2.30	0.89	2.29	0.98	59.454	0.22
Sexist traits associated with F/M	2.10	0.78	2.07	0.84	70.725	0.24
Ability of each sex to perform roles and functions	1.60	0.75	1.33	0.65	156.573 ***	0.36 ***

*** *p* < 0.001.

**Table 4:** Descriptive analysis moral disengagement questionnaire.

	Male Victims		Female Victims			
	$\overset{-}{x}$	SD	$\overset{-}{x}$	SD	*χ^2^*	*V*
Moral justification	2.01	0.55	1.68	0.45	142.63 ***	0.342 ***
Euphemistic language	1.37	0.38	1.16	0.28	131.72 ***	0.329 ***
Advantageous comparison	1.63	0.49	1.44	0.38	63.18 ***	0.228 ***
Diffusion of responsability	1.59	0.46	1.55	0.43	16.23 **	0.115
Distorsión of consequences	1.42	0.37	1.28	0.29	52.829 ***	0.208 ***
Ascription of blame	1.52	0.40	1.38	0.34	58.759 ***	0.220 ***
Dehumanization	1.53	0.50	1.33	0.40	82.414 ***	0.260 ***

** *p* < 0.01; *** *p* < 0.001.

**Table 5:** Analysis of mean differences.

	*T*	gl	*p*	Mean Differences
Total violence incidence	1.76	1097.543	0.08	0.03
Total perception of severity	13.31	1015.358	0.000	0.73
Hostile sexism	6.39	1072.025	0.000	0.26
Benevolent sexism	0.25	1174.757	0.79	0.01
Sexist traits associated with F/M	0.013	1179.845	0.99	0.06
Attitudes of each sex to performance of roles	6.42	1040.804	0.000	0.30
Moral justification	12.01	1032.755	0.000	0.34
Euphemistic language	10.44	894.995	0.000	0.22
Advantageous comparison	6.95	1031.426	0.000	0.17
Diffusion of responsability	2.22	1045.414	0.02	0.06
Distorsión of consequences	6.49	900.854	0.000	0.13
Victims blaming	7.22	966.027	0.000	0.15
Dehumanization	7.77	962.802	0.000	0.21

**Table 6:** Linear regression.

Model 1: Male						Model 2: Female					
			R	R^2^	F				R	R^2^	F
1	Hostile sexism	0.205 ***	0.042	0.040	21.455	1	Instrumental violence severity	−0.221 ***	0.049	0.047	34.847
2	Hostile sexism	0.177 ***	0.068	0.065	17.864	2	Instrumental violence severity	−0.211 ***	0.084	0.081	31.137
	Negative emotions	0.164 ***					Negative emotions	0.188 ***			
3	Hostile sexism	0.148 ***	0.079	0.073	13.863	3	Instrumental violence severity	−0.340 ***	0.098	0.094	24.583
	Negative emotions	0.166 ***					Negative emotions	−191 ***			
	Instrumental violence severity	−0.106 *					Detachment severity	0.176 ***			
4	Hostile sexism	0.162 ***	0.092	0.085	12.302	4	Instrumental violence severity	−0.340 ***	0.11	0.105	20.915
	Negative emotions	0.158 ***					Negative emotions	0.161 ***			
	Instrumental violence severity	−0.236 ***					Detachment severity	0.184 ***			
	Detachment severity	0.177 ***					Moral justification	0.114 **			
5	Hostile sexism	0.163 ***	0.11	0.096	11.327						
	Negative emotions	0.157 ***									
	Instrumental violence severity	−0.138 *									
	Detachment severity	0.295 ***									
	Humiliation severity	−0.232 **									
6	Hostile sexism	0.172 ***	0.12	0.104	10.424						
	Negative emotions	0.153 ***									
	Instrumental violence severity	−0.170 *									
	Detachment severity	0.242 **									
	Humiliation severity	−0.339 ***									
	Coercion severity	0.209 *									

* *p* < 0.05; ** *p* < 0.01; *** *p* < 0.001.

## Data Availability

Data not available due to privacy.
